# Structure and Mixed Proton–Electronic Conductivity in Pr and Nb-Substituted La_5.4_MoO_12−δ_ Ceramics

**DOI:** 10.3390/ma18030529

**Published:** 2025-01-24

**Authors:** Abraham Sánchez-Caballero, José M. Porras-Vázquez, Lucía dos Santos-Gómez, Javier Zamudio-García, Antonia Infantes-Molina, Jesús Canales-Vázquez, Enrique R. Losilla, David Marrero-López

**Affiliations:** 1 Departamento de Química Inorgánica, Cristalografía y Mineralogía, Universidad de Málaga, 29071 Málaga, Spain; abraham11sc@uma.es (A.S.-C.); ldsg@uma.es (L.d.S.-G.); ainfantes@uma.es (A.I.-M.); r_losilla@uma.es (E.R.L.); 2 Departmento of Energy Conversion and Storage, Technical University of Denmark, Fysikvej, 310, 2800 Kongens Lyngby, Denmark; javzam@dtu.dk; 3 Renewable Energy Research Institute, Escuela Técnica Superior de Ingenieros Industriales de Albacete, University of Castilla-La Mancha, 02071 Albacete, Spain; jesus.canales@uclm.es; 4 Departamento de Física Aplicada I, Universidad de Málaga, 29071 Málaga, Spain

**Keywords:** La_5.4_MoO_12-δ_, polymorphism, mixed ionic–electronic conductivity

## Abstract

Lanthanide molybdates are materials known for their mixed proton–ionic conductivity. This study investigates the effects of Pr content and Nb-doping on the crystal structure and electrical properties of the La_5.4−x_Pr_x_Mo_1−y_Nb_y_O_12−δ_ (x = 0, 1.35, 2.7, 4.05, 5.4; y = 0, 0.1) series. The research focuses on two primary objectives: (i) enhancing the electronic conductivity through the use of Pr^4+^/Pr^3+^ redox pairs and (ii) increasing the ionic conductivity through Nb^5+^ aliovalent doping. The materials were thoroughly characterized by X-ray powder diffraction (XRD), X-ray photoelectron spectroscopy (XPS), transmission and scanning electron microscopy (TEM and SEM), and complex impedance spectroscopy. The average crystal structure of the materials depended significantly on the Pr content. In general, compositions with a higher Pr content crystallize in a cubic fluorite-type structure, whereas those with a lower Pr content stabilize a rhombohedral polymorph. However, detailed TEM studies reveal a more complex local crystal structure characterized by nanodomains and incommensurate modulations. The highest conductivity values were observed in a N_2_ atmosphere for compositions with an elevated Pr content, with values of 0.17 and 204.4 mS cm^−1^ for x = 0 and x = 5.4, respectively, at 700 °C, which is attributed to electronic conduction mediated by the Pr^4+^/Pr^3+^ redox pair, as confirmed by XPS. These findings highlight the potential of tailored doping strategies to optimize the conducting properties of lanthanide molybdates for specific high-temperature electrochemical applications.

## 1. Introduction

High-temperature proton-conducting ceramics have a wide range of industrial applications due to their exceptional thermal stability, chemical resistance, and mechanical strength. They are commonly used as gas sensors, electrolytes in solid oxide fuel cells and electrolyzers, and membranes for hydrogen separation and purification [1,2,3,4,5].

BaCeO_3_ and BaZrO_3_ perovskites are among the most commonly studied material membranes for hydrogen separation due to their high-proton conductivity [6,7,8]. However, BaCeO_3_ suffers from low chemical stability in H_2_O and CO_2_ environments, while BaZrO_3_, despite its improved stability, requires very high sintering temperatures to achieve the desired level of densification. To combine the properties of both materials, BaCe_1−x_Zr_x_O_3_ compositions have been investigated. These materials have been optimized through appropriate doping with Yb and Y, with the composition BaZr_0.2_Ce_0.6_Y_0.1_Yb_0.1_O_3_ showing the highest proton conductivity values for fuel cell application [9]. However, the high basicity of these materials remains a drawback for their application in CO_2_-rich environments [10]. For this reason, alternative proton conductors without alkaline earth elements, such as La_2_Ce_2_O_7_ and LaNbO_4_, have been investigated [11,12,13].

Lanthanum tungstates, La_6−x_WO_12−δ_, have emerged as promising proton-conducting materials with tunable properties for both fuel cell electrolytes and ceramic hydrogen separation membranes due to their chemical stability, as well as their resistance to gasses such as H_2_S, CO, and CO_2_ [14,15,16,17]. A strategy for enhancing the electronic conductivity of these materials consists of partially or completely substituting tungsten with molybdenum, resulting in La_6−x_MoO_12−δ_ compounds, which exhibit higher electronic conductivity due to the easier reducibility of Mo^6+^ compared to W^6+^ [18,19,20].

In recent years, our research group has investigated the relationship between composition and synthesis-sintering conditions in lanthanum molybdates, leading to tunable structural and electrical properties [21,22]. The La_6−x_MoO_12−δ_ series with a different La-content (0 ≤ x ≤ 3) was synthesized using the freeze-drying method, where the precursors were heated to 1500 °C and subsequently cooled at different rates. Samples that were rapidly cooled exhibited a disordered cubic fluorite structure. However, at lower cooling rates and for high lanthanum contents (x ≤ 1), two differently ordered rhombohedral polymorphs were observed: one with a 7 × 7 × 1 supercell relative to disordered cubic fluorite denoted as R1 and another with a 5 × 5 × 1 supercell denoted as R2. The crystal structures were satisfactorily determined using a combination of HRTEM images and SAED patterns, employing De Wolff’s theory and the Rietveld method with neutron powder diffraction data. For lower lanthanum contents, 1.5 ≤ x ≤ 3, the cubic symmetry was favoured at high sintering temperatures (1400–1500 °C) and rapid cooling (5 °C min^−1^), while a monoclinic polymorph [23] was obtained at lower sintering temperatures (1100–1300 °C) and very slow cooling (0.5 °C min^−1^).

The preparation conditions and composition significantly influence the electrical properties of lanthanum molybdates. A decrease in ionic conductivity was observed as the La/Mo ratio decreased; however, this was accompanied by an increase in electronic conductivity, which was significantly enhanced under reducing conditions (5% H_2_-Ar) [24]. Furthermore, the symmetry and electrical properties of the samples can be tailored by the aliovalent substitution of Mo^6+^ by Nb^5+^, resulting in the stabilization of the R1 polymorph and improved ionic conductivity due to the generation of additional oxide ion vacancies [24].

Shlyakhtina et al. [25,26] also studied the structural and electrical properties of molybdates with lanthanides smaller than lanthanum, Ln_5.5_MoO_12−δ_, which exhibit different symmetries depending on the ionic radius of the lanthanide. They observed a fluorite-type cubic structure for Ln = Sm^3+^, Gd^3+^, and Dy^3+^ (s.g. $Fm\bar{3}m$) and a cubic bixbiite type for Ln = Ho^3+^, Er^3+^, Tm^3+^, Yb^3+^, and Lu^3+^ (s.g. $Ia\bar{3}$), noting a decrease in total conductivity as the size of the lanthanide decreased. In our research group, we also conducted a comprehensive investigation into the relationship between structural and electrical properties and synthesis-sintering conditions for middle-sized lanthanides, Ln_5.4_MoO_11.1_ (Ln = Nd^3+^, Sm^3+^ and Gd^3+^) [27]. With the decreasing lanthanide size, cubic symmetry tends to dominate even at extremely low cooling rates due to reduced size differences between the lanthanide and molybdenum. Electrical property analysis also revealed lower ionic conductivity for smaller lanthanides but a significant enhancement in the electronic conductivity under reducing conditions.

Continuing the research on this family of proton conductors, the primary aim of this study is to investigate the influence of Pr-doping in La_5.4_MoO_11.1_ to enhance electronic conductivity. This is motivated by the known effect of the Pr^4+^/Pr^3+^ pair in conferring significant electronic conductivity in energy-applied materials [28,29]. Additionally, it has been reported that Nb^5+^-doping at the W^6+^/Mo^6+^ site in lanthanum tungstates/molybdates creates oxide vacancies, thereby enhancing ionic conductivity [24,30]. Therefore, in this work, we aim to improve the ionic–electronic conductivity of lanthanum molybdates through this synergistic approach. The materials were thoroughly characterized using X-ray powder diffraction, X-ray photoelectron spectroscopy, transmission and scanning electron microscopy and impedance spectroscopy under different gas-flowing atmospheres to evaluate the materials’ potential as ceramic membranes for hydrogen separation.

## 2. Materials and Methods

### 2.1. Synthesis

La_5.4−x_Pr_x_Mo_1−y_Nb_y_O_12−δ_ samples (x = 1.35, 2.7, 4.05, 5.4; y = 0, 0.1) were synthesized using a freeze-drying precursor method, following the synthetic procedure described in a previous study for related compositions [21]. The starting reagents used were La_2_O_3_ (99.99%, Merck, Darmstadt, Germany), Pr_6_O_11_ (99.9%, Merck), MoO_3_ (99.5%, Merck), and Nb(HC_2_O_4_)_5_·H_2_C_2_O_4_ (97%, ABCR, Karlsruhe, Germany). La_2_O_3_ and Pr_6_O_1_ salts were dissolved in diluted nitric acid, while MoO_3_ was dissolved separately in diluted ammonia, and Nb(HC_2_O_4_)_5_·H_2_C_2_O_4_ was dissolved in water. After dissolution, an Ethylenediaminetetraacetic acid (EDTA 99.99%, Merck) solution was added as a chelating agent in a 1:1 metal-to-EDTA ratio to both solutions, and the pH was adjusted to approximately 7 by adding ammonia. The cation solutions were mixed under stirring to obtain transparent solutions with a concentration of approximately 0.1 mol L^−1^ and a pH of 7.

The solutions were frozen in liquid nitrogen and freeze-dried in Scanvac-Coolsafe equipment for 2 days. The precursor powders were initially heated at 300 °C for 1 h, followed by calcination at 800 °C for 1 h to completely remove any remaining carbonaceous residues. The resulting powders were pressed into pellets with a diameter of 10 mm and a thickness of 1 mm and then sintered at 1500 °C for 1 h with a heating rate of 10 °C min^−1^, followed by cooling at three different rates: quenching (Q), 5, and 0.5 °C min^−1^. The choice of the cooling rate is critical as it can tailor the final crystal symmetry and electrical properties of the samples. For structural analysis, the pellets were ground into fine powders. All samples are designated as La_5.4−x_Pr_x_Nb_y__C, where x and y represent the Pr and Nb content, respectively, and C denotes the cooling rate employed.

### 2.2. Structural and Microstructural Characterization

The crystal structure of the samples was analyzed using laboratory X-ray powder diffraction with a PANalytical Empyrean diffractometer equipped with CuKα_1,2_ radiation and X’Pert Pro MPD (PANalytical, Almelo, The Netherlands) with CuKα_1_ radiation. The measurements were collected over a 2θ angular range of 10–80° with an acquisition time of 1 h. Phase identification was performed using X’Pert HighScore Plus software [31], and structural analysis was carried out with GSAS software [32].

X-ray photoelectron spectra were collected using a Scienta ESCA 200 spectrometer equipped with an X-ray Al(Kα) source (1486.6 eV), operating under an ultra-high vacuum of 10^−10^ mbar. The experimental procedure for XPS analysis utilized a full width at half maximum of 0.65 eV for the Au 4*f*_7/2_ line. Data analysis was performed using the Multipak-V9.3 software [33]. A Shirley-type background was applied to the signals, and the spectra were fitted with Gaussian−Lorentzian curves to precisely determine the binding energy of the element core levels.

Selected area electron diffraction (SAED) and high-resolution transmission electron microscopy (HRTEM) were employed to study the crystal structure of the materials. TEM specimens were prepared by suspending finely ground powders in acetone and depositing a drop of the mixture onto a holey-carbon Cu grid (EMS). The analysis was performed using a Jeol JEM 2100 electron microscope operating at 200 kV (Akishima, Japan), equipped with a ±25° double-tilt sample holder. Images were analysed using the Digital Micrograph™ software from Gatan [34].

The morphology and cation composition of the ceramic pellets were examined using field emission scanning electron microscopy (FEI-SEM, Helios Nanolab 650, Thermo Fischer Scientific, Waltham, MA, USA) coupled with energy dispersive spectroscopy (EDS, X-Max Oxford Instruments, Oxford, UK). The grain size was determined from the SEM micrographs using the linear intercept method [35].

### 2.3. Electrical Characterisation

Impedance spectroscopy was used to measure the electrical conductivity, employing a frequency response analyser (Solartron 1260 FRA, Hampshire, UK) under dry and wet N_2_ (2 vol.% H_2_O) and wet 5% H_2_/Ar atmospheres. The measurements were performed over a frequency range of 1 MHz to 0.01 Hz with an AC amplitude of 50 mV during cooling from 750 to 100 °C, with a 30 min dwell time for each temperature. Platinum current collectors were prepared by coating Pt-ink (METALOR^®^ 6082, Marin, Switzerland) to the pellet surfaces, followed by firing at 800 °C for 1 h in the air. Data analysis was carried out using ZView software [36].

## 3. Results

### 3.1. Phase Formation

**Figure 1:**
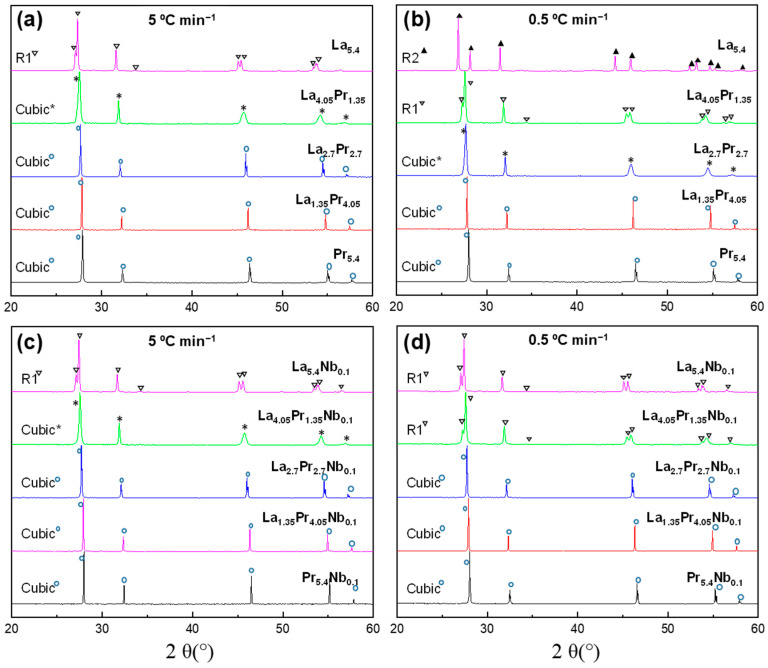
XRD patterns for La_5.4−x_Pr_x_Mo_1−y_Nb_y_O_12−δ_ (La_5.4−x_Pr_x_) samples (x = 1.35, 2.7, 4.05, 5.4; y = 0, 0.1) heated at 1500 °C and cooled down at (**a**,**c**) 5 °C min^−1^ and (**b**,**d**) 0.5 °C min^−1^. The symmetry of the samples is indicated within the figure: cubic ($Fm\bar{3}m$) and rhombohedral ($R\bar{3}$) for both R1 and R2 polymorphs. Several samples (*) exhibit broad diffraction peaks, suggesting a deviation from cubic symmetry. The peak splitting observed in some cubic polymorphs at high angles is due to CuKα_2_ contribution.

X-ray diffraction patterns of the La_5.4−x_Pr_x_Mo_1−y_Nb_y_O_12−δ_ series, heated at 1500 °C for 1 h and subsequently cooled at different rates, are shown in Figure S1 (quenched samples, Q) and Figure 1 (5 and 0.5 °C min^−1^ cooling rates). All the samples are single-phase compounds; however, the crystal symmetry is dependent on both the composition and cooling rate employed. Mo volatilization is considered negligible in these compounds, as reported previously for related compositions, based on EDS and Inductively Coupled Plasma measurements [37].

All quenched samples (Q), regardless of the composition, were crystallized in a disordered cubic fluorite, similar to La_6−x_Mo_1−y_Nb_y_O_12−δ_ (x = 0.6–3; y = 0–0.2) [21,22,24] and Ln_5.4_MoO_11.1_ (Ln = Nd, Sm and Gd) series [27], which were also rapidly cooled from 1500 °C. This behaviour can be attributed to the high thermal vibration of the atoms at 1500 °C, causing them to occupy the same atomic positions: (0, 0, 0) for the cations (La, Pr, Mo and Nb) and (¼, ¼, ¼) for the oxide anions, all within the $Fm\bar{3}m$ space group. Consequently, the quenching process preserves these atomic positions at room temperature, resulting in a high-symmetry cubic fluorite structure.

At lower cooling rates, such as 5 and 0.5 °C min^−1^, thermodynamic factors become predominant, favouring the stabilization of the R1 rhombohedral polymorphs. This behaviour can be attributed to the varying ionic radii of the cations: 1.16, 1.02, 1.13, 0.96. and 0.74 Å for La^3+^, Mo^6+^, Pr^3+^, Pr^4+^, and Nb^5+^, respectively, all in an eightfold coordination. This size mismatch, along with the lower cooling rates, allows the cations to settle into thermodynamically stable positions, leading to a greater distortion of the coordination spheres and, consequently, a decrease in symmetry. Under these cooling conditions, the cubic symmetry is stabilized as the praseodymium content increases (Figure 1) due to the smaller size of Pr^4+^ and Pr^3+^ compared to La^3+^, which reduces distortions in the molybdenum coordination sphere. The transition from the rhombohedral to the cubic polymorph is gradual with the increasing Pr content. However, La_4.05_Pr_1.35__5, despite having an average cubic structure, shows broad diffraction peaks, suggesting some rhombohedral distortion. In contrast, La_2.7_Pr_2.7__5 exhibits a cubic structure with narrow diffraction peaks. For the samples cooled down at 0.5 °C min^−1^, where thermodynamic effects are more pronounced, a higher Pr content is required to stabilize the cubic phase. La_4.05_Pr_1.35__0.5 can still be considered R1, and La_2.7_Pr_2.7_0.5 also shows a significant rhombohedral distortion. Complete conversion to the cubic phase is only achieved for La_1.35_Pr_4.05__0.5.

Nb-doping also plays an important role in determining the final symmetry of the samples (Figure 1c,d). For instance, as mentioned earlier, La_2.7_Pr_2.7__0.5 exhibits a rhombohedral distortion, whereas La_2.7_Pr_2.7_Nb_0.1__0.5, cooled at the same rate, crystallizes into a simple cubic fluorite structure with narrow diffraction peaks. A similar behaviour was observed in previous work for La_5.4_MoO_11.1_ [24], where the undoped sample, cooled down at 0.5 °C min^−1^, exhibited an R2 symmetry, while 10% Nb-doping at the molybdenum site stabilized the R1 structure. This effect is likely due to the generation of oxide vacancies induced by aliovalent doping, which influences the structural stability and symmetry of the materials.

### 3.2. Structural Analysis

The structural characterization of all samples was conducted using the Rietveld method. For the cubic compositions, a simple fluorite structural model was employed ($Fm\bar{3}m$ space group), where all cations (La, Mo, Pr and Nb) share the same crystallographic position, all of them with an eightfold coordination. Occupancy factors were adjusted to match the theoretical compositions, and isotropic atomic displacement factors were constrained to a single value for consistency. The refinement process included fitting the unit cell, scale factor, background, peak shape coefficients, and isotropic displacement parameters.

For the rhombohedral samples, which exhibit more complex crystal structures, a Le Bail analysis was performed using an $R\bar{3}$ space group. The structural models previously reported based on neutron powder diffraction [22] could not be used for X-ray diffraction studies, as X-ray radiation is less sensitive to oxygen ordering, which is critical for stabilizing the different polymorphs observed in these samples. Nonetheless, all cation environments in those models also have an eightfold coordination.

**Figure 2:**
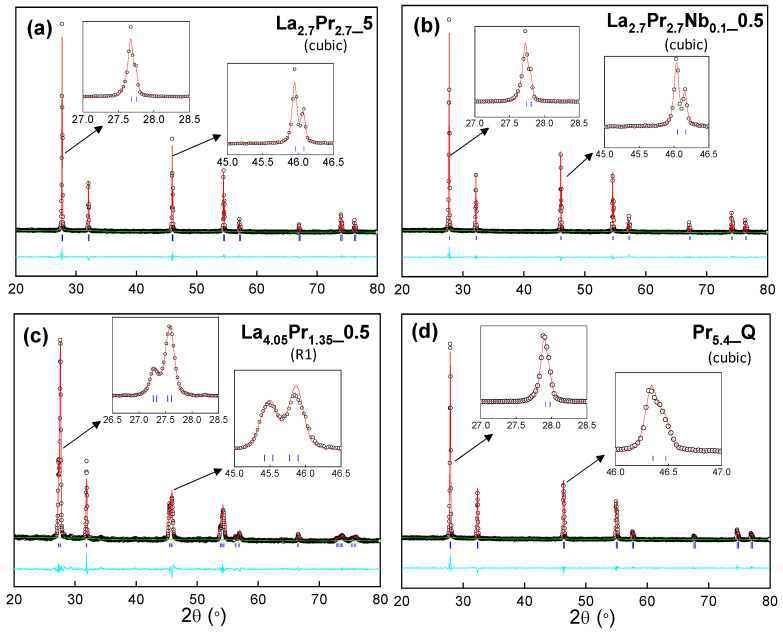
XRD Rietveld plots for (**a**) La_2.7_Pr_2.7__5, (**b**) La_2.7_Pr_2.7_Nb_0.1__5, (**c**) La_4.05_Pr_1.35__0.5, and (**d**) Pr_5.4__Q. Crystal system and agreement factors are displayed within the figure. [Observed data (crosses), calculated pattern (red continuous line), difference curve (cyan line) and reflection marks (blue short lines)]. The inset figures highlight the details of several diffraction peaks for both cubic and R1 polymorphs.

In general, the refinements exhibit very low disagreement factors, typically in the range of 5–12% for R_ap_ and 1–5% for R_F_ (Figure 2 and Table S1). Regardless of the cooling rate, the cell parameters decrease as the Pr content increases, with cell volume values of 45.51(1), 43.87(1), and 42.20(1) Å^3^ for La_5.4__Q, La_2.7_Pr_2.7__Q and Pr_5.4__Q, respectively. This reduction in cell volume is attributed to the smaller size of Pr^3+^/Pr^4+^ (1.13 and 0.96 Å) compared to La^3+^ (1.16 Å). Additionally, Nb-doping results in decreased cell volumes for all cooling rates due to the smaller size of Nb^5+^ (0.74 Å) compared to Mo^6+^ (1.02 Å). For instance, La_2.7_Pr_2.7__Q and La_2.7_Pr_2.7_Nb_0.1__Q exhibit cell volumes of 43.87(1) and 43.67(1) Å^3^, respectively. Finally, for a given composition, the highest cell volume is consistently observed for the quenched samples, with values of 43.87, 43.57, and 43.58 Å^3^ for La_2.7_Pr_2.7__Q, La_2.7_Pr_2.7__5, and La_2.7_Pr_2.7__0.5, respectively. This behaviour is possibly caused by the higher reducibility of Pr^4+^ at elevated temperatures, leading to a higher amount of Pr^3+^ and, thus, an increase in the cell volumes. Conversely, at lower cooling rates, the formation of Pr^4+^ is favoured, resulting in decreased cell parameters.

After a reduction in the samples in a 5% H_2_-Ar flowing atmosphere at 800 °C for 24 h, most of the Pr^4+^ is potentially reduced to Pr^3+^, resulting in an increase in cell volume that shows minimal variation regardless of the cooling rate. For instance, Pr_5.4__Q, Pr_5.4__5, and Pr_5.4__0.5 exhibit values after reductions of 42.91(1), 42.92(1), and 42.94(1) Å^3^, respectively, which is higher than those for the as-prepared samples at 42.34(1), 42.03(1), and 41.99 (1) Å^3^.

**Figure 3:**
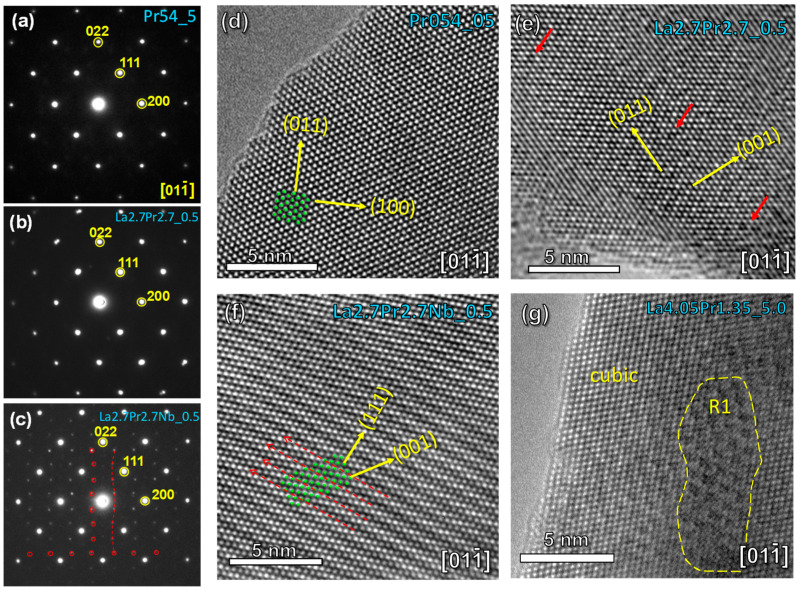
SAED patterns of (**a**) Pr_5.4__5, (**b**) La_2.7_Pr_2.7__0.5, and (**c**) La_2.7_Pr_2.7_Nb_0.1__0.5 in the $\left[01\bar{1}\right]$ zone axis. HRTEM images of (**d**) Pr_5.4__0.5, (**e**) La_2.7_Pr_2.7__0.5, (**f**) La_2.7_Pr_2.7_Nb_0.1__0.5, and (**g**) La_4.05_Pr_1.35__5 in the $\left[01\bar{1}\right]$ zone axis.

The local crystal structure was further examined using both electron diffraction (SAED) and HRTEM techniques. Figure 3a–c provides a comparative analysis of the SAED patterns obtained for selected compositions along the $\left[1\bar{1}0\right]$ zone axis. Samples with a high Pr content (Pr_5.4__5) exclusively present reflections corresponding to the cubic fluorite structure (Figure 3a). This identical SAED pattern persists in samples with a lower Pr content, such as La_2.7_Pr_2.7__0.5 (Figure 3b), despite the broad diffraction peaks observed by XRD (Figure 1).

Conversely, a completely different atomic arrangement is observed for La_2.7_Pr_2.7_Nb_0.1__0.5 after Nb-doping (Figure 3c). The SAED patterns for these samples reveal additional diffraction reflections along specific crystallographic directions, notably [200] and [022]. This observation is consistent with an incommensurate modulation, as evidenced in previous studies using neutron diffraction data for related compositions [24]. Such an incommensurate structure is typically attributed to the local ordering of oxygen vacancies, likely induced by the aliovalent Nb-doping.

The HRTEM images corroborate the findings derived from the SAED analysis (Figure 3d–g). Pr_5.4_0.5 exhibits an atomic arrangement consistent with a defect-free cubic fluorite structure (Figure 3d). However, as the Pr content decreases, numerous local defects (marked by red arrows) become apparent, as observed in La_2.7_Pr_2.7__0.5 (Figure 3e). This defect-rich structure transforms into a modulated arrangement with Nb-doping due to the higher concentration of oxygen vacancies (Figure 3f). Finally, in the sample with the lowest Pr content, La_4.05_Pr_1.35__5, a predominantly cubic fluorite structure can be observed, interspersed with embedded microdomains of the R1 polymorph (Figure 3g). These microdomains explain the broad diffraction peaks observed in XRD patterns and the gradual symmetry transition observed as the Pr content increases (Figure 1).

### 3.3. XPS Analysis

**Figure 4:**
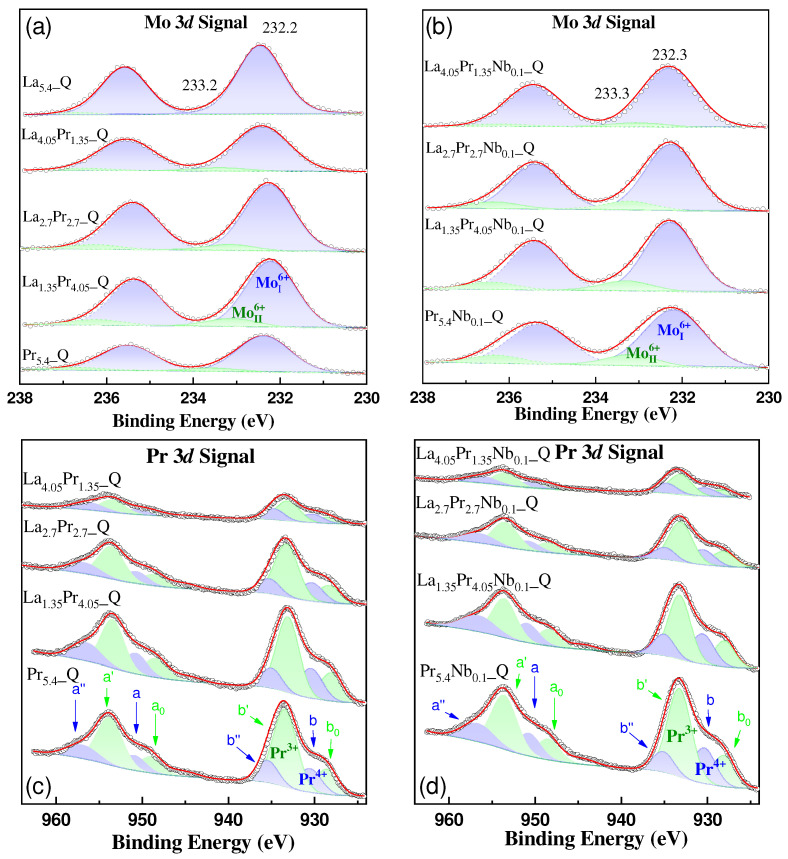
XPS spectra of (**a**,**b**) Mo 3*d* and (**c**,**d**) Pr 3*d* core levels for the La_5.4−x_Pr_x_Mo_1−y_Nb_y_O_12−δ_ (x = 1.35, 2.7. 4.05, 5.4; y = 0, 0.1) series.

The surface chemical state and atomic concentration information of the quenched samples in the air were obtained from X-ray photoelectron spectroscopy. The Mo 3*d* core level spectra (Figure 4a,b) consist of two doublets, Mo 3*d*_5/2_ and Mo 3*d*_3/2_. The predominant component, Mo 3*d*_5/2_, is located at 232.2 eV, characteristic of the Mo^6+^ species, as reported previously [21]. Additionally, a minor contribution at a higher binding energy (BE) of 233.3 eV is detected and can also be attributed to the Mo^6+^ species but in different chemical environments. After Nb doping, the same contributions are observed in the Mo3*d* core level spectra, with the higher BE component at 233.3 eV becoming more prominent, especially in samples with a higher Pr content. This indicates that in these samples, the crystal symmetry is more susceptible to alteration due to Nb incorporation.

The Pr 3*d* spectra provide information about the Pr^4+^/Pr^3+^ ratio present in the samples (Figure 4c,d). The deconvolution of the spectra, as is common for many lanthanides, reveals several electron satellite structures for the 3*d* and 4*d* ionizations. According to Poggio-Fraccari et al. [38], three doublets (blue lines) are assigned to Pr^4+^ (called a”-b”, a-b), and two doublets (green lines) are assigned to Pr^3+^ (called a’-b’, a_o_-b_o_). Therefore, in all cases, both Pr^4+^ and Pr^3+^ species are present. The corresponding atomic ratios are included in Table S2, which shows that the Pr^4+^/Pr^3+^ ratio becomes smaller as the Pr content increases and remains practically stable after Nb incorporation.

Similarly, to Pr, the lanthanum spectra are also complex. Figure S2a,b show the La 3*d*_5/2_ component for the different samples with and without Nb-doping. In all cases, four contributions are clearly distinguishable. The main peak, centred at 834.7 eV, is assigned to La^3+^ related to La−Mo−O mixed oxides [39]. The grey dashed lines correspond to satellite peaks. A second much less intense contribution is located at approximately 833.0 eV, observed in all cases. Its intensity is directly related to the La and Pr content as well as the presence of Nb. As previously reported, this is assigned to a new chemical environment for La^3+^, similar to what was observed in the Mo 3*d* signal. Therefore, the proportion of these species increases with both the Pr content and after Nb addition.

Finally, the O 1*s* signal, which is much more complex, is also included in Figure S2c,d, where four contributions are noticeable. For samples without Nb-doping, the Pr_5.4__Q sample shows contributions at 528.5, 530.2, 531.5, and 532.6 eV (Figure S2c), which are associated with a rhombohedral polymorph containing domains of different symmetry as confirmed by TEM studies and previously observed in the Mo and La signals [27]. Specifically, these contributions correspond to the O-lattice related to MoO_3_ (∼530.9 eV), Pr^3+^ (∼531.9 eV) [40], defective oxygen close to oxygen vacancies (531.1–531.4 eV) [41], surface hydroxyl oxygen species, and adsorbed oxygen, respectively.

After Pr substitution with La, the contributions at approximately 528.5 and 530.2 eV become more significant due to a partial loss of symmetry (Figure S2d). This change is also observed in the Mo and La signals, as well as the presence of La oxide, which has a BE of 530.5 eV. At higher La contents, the contribution at 528.5 eV becomes almost negligible as the cubic symmetry is again recovered. Therefore, the La_5.4_Q sample mainly shows two contributions: one at 530.7 eV, associated with the presence of molybdenum, and another at 532.0 eV, corresponding to OH groups and adsorbed oxygen.

On the other hand, the incorporation of Nb into the structure results in a loss of symmetry, regardless of the sample composition, as observed from the Mo and La signals. This is evident from the increase in the O1s signal at 528.5 eV, which is particularly pronounced at a higher Pr content.

### 3.4. Microstructural and Electrical Characterization

**Figure 5:**
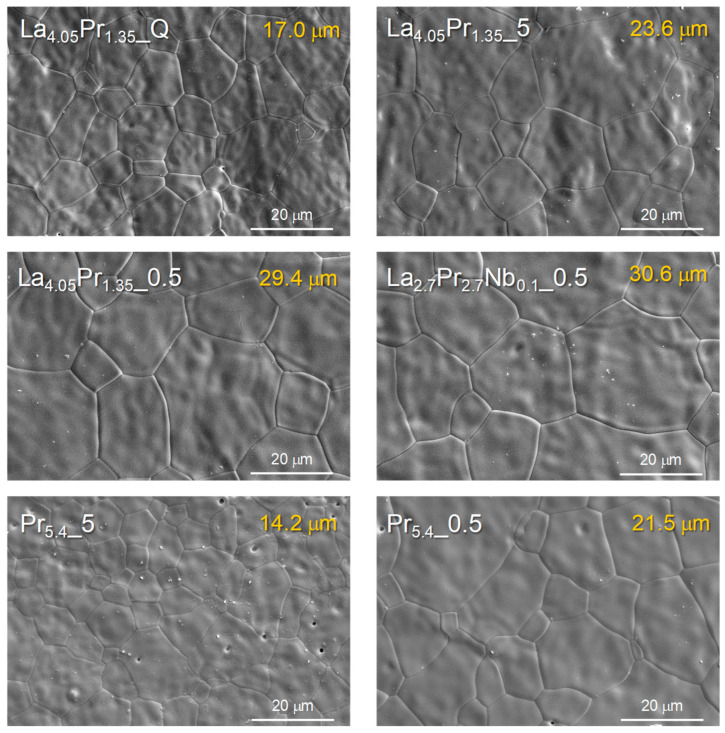
SEM micrographs of the La_5.4−x_Pr_x_Mo_1−y_Nb_y_O_12−*δ*_ samples (La_5.4−x_Pr_x_Mo_1−y__C) (x = 1.35, 2.7. 4.05, 5.4; y = 0, 0.1) sintered at 1500 °C for 1 h and cooled down at different rates. Average grain size is denoted at the top right of the images.

SEM images reveal that all ceramic materials are nearly fully dense (Figure 5), with a relative density close to 100%. No detectable fractures, secondary phases, or visible segregations at the grain boundaries are observed for the different materials. These findings are further confirmed by EDS analysis, which shows homogenous cation distribution for La_2.7_Pr_2.7__Q as a representative sample (Figure S3). Similar elemental mappings can be observed for the remaining samples, regardless of the composition or cooling rate.

The analysis of the average particle size reveals that, in general, higher Pr contents lead to a decrease in grain size, with values of 23.6, 20.7, and 14.2 μm for La_4.05_Pr_1.35__5, La_2.7_Pr_2.7__5, and Pr_5.4__5, respectively. A similar trend was observed in previous work, where molybdates containing smaller lanthanides exhibited reduced average grain sizes [27]. On the other hand, Nb-doping does not appear to have a significant effect on the particle size. Additionally, the average grain size increases as the cooling rate decreases, with values of 11.8, 14.2, and 21.5 μm for Pr_5.4__Q, Pr_5.4__5, and Pr_5.4__0.5, respectively. This increase is attributed to the longer dwelling time at 1500 °C for the lower cooling rates, which allows for more extensive grain growth.

**Figure 6:**
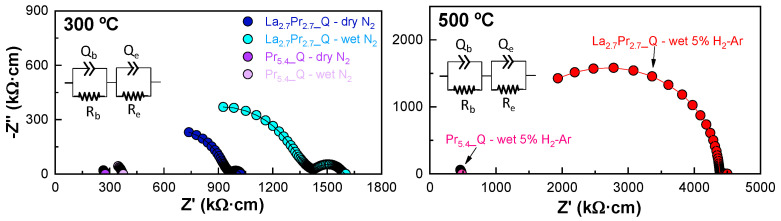
Representative impedance spectra of La_2.7_Pr_2.7__0.5 and Pr_5.4__Q under (**left** dry/wet N_2_ at 300 °C and (**right**) 5% H_2_-Ar gasses at 500 °C. The equivalent circuit used to fit the spectra is shown in the insets.

Figure 6 shows the impedance spectra for La_2.7_Pr_2.7__Q and Pr_5.4__Q as representative examples of the series, both quenched and measured under dry/wet N_2_ and wet 5% H_2_−Ar (similar plots were obtained for the other compositions). The plots reveal the presence of two different contributions: one corresponding to bulk conduction within the grain interior, characterized by capacitances on the order of 10^−12^ F cm^−1^, and the other associated with electrode processes, characterized by capacitances on the order of 10^−2^ F cm^−1^ [21]. These plots can be accurately fitted using an (R*_b_*Q*_b_*)(R*_e_*Q*_e_*)-equivalent circuit model, where the subscripts *b* and *e* denote the bulk and electrode processes, respectively (inset Figure 6). It is important to note that no grain boundary contribution to the overall conductivity was observed, likely due to the large average grain size and the absence of secondary phases, as confirmed by SEM images.

Notably, under wet N_2_ conditions, the bulk resistance increases compared to a dry N_2_ atmosphere. This contrasts with findings from other studies on La-, Sm-, and Gd-based molybdates [21,22,27], where water incorporation into the oxide vacancies enhanced proton conductivity. However, this behaviour is similar to that observed in Nd_5.4_MoO_11.1_ [27] and in other Nd-containing proton conductors, such as Nd-doped La_2_Ce_2_O_7_ [42], where an increase in p-type conductivity was observed with increasing Nd content. This phenomenon was attributed to the fact that Nd^3+^ can oxidize to Nd^4+^, although it is less prone to oxidation compared to Pr^3+^/Pr^4+^. Therefore, the significant p-type electronic contribution to the overall conductivity in Pr containing molybdates suggests a competition between O_2_ and H_2_O for absorption into the oxide vacancies, which reduces the proton conductivity.

**Figure 7:**
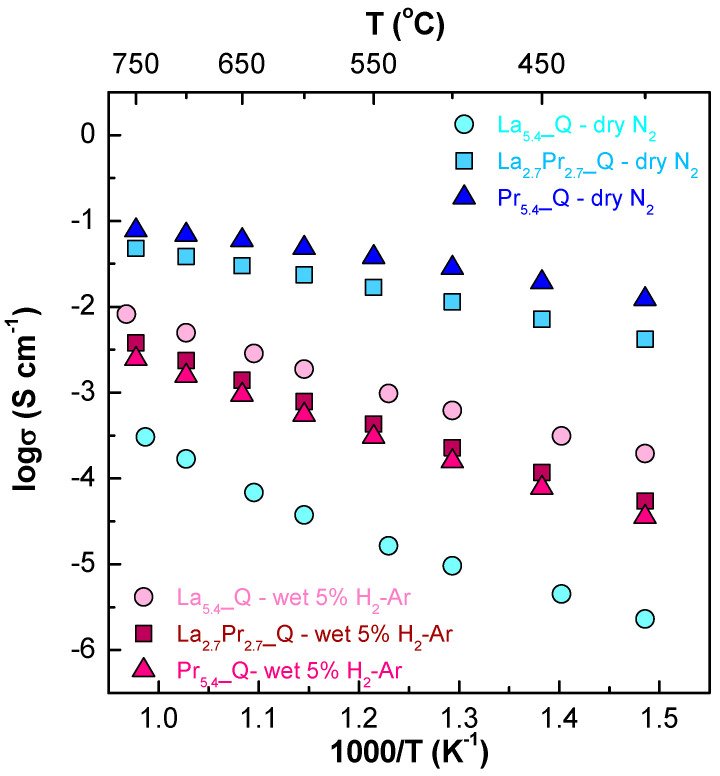
Arrhenius plots of La_5.4_Q, La_2.7_Pr_2.7__Q, and Pr_5.4__Q sintered at 1500 °C for 1 h and cooled down by quenching under dry N_2_ and wet 5% H_2_-Ar atmospheres.

The Arrhenius plots (Figure 7) and Table S3 clearly demonstrate that in a N_2_ atmosphere, the conductivity increases significantly with higher Pr contents, ranging from 0.17 to 204.4 mS cm^−1^ for La_5.4__Q and Pr_5.4__Q, respectively, at 700 °C. This increase is attributed to the presence of the Pr^4+^/Pr^3+^ pair, which enhances the electronic contribution to the overall conductivity. Furthermore, the activation energies decrease significantly as the Pr content increases, with values of 1.04(1), 0.37(1), and 0.26(1) eV for La_5.4__Q, La_2.7_Pr_2.7__Q, and Pr_5.4__Q, respectively, in the 750–550 °C temperature range under dry N_2_. This behaviour suggests a transition in the transport properties from predominantly ionic conduction for samples with a low Pr content to predominantly electronic conduction in those with a high Pr content. Therefore, the mixed ionic–electronic conductivity of these materials can be tailored through appropriate Pr substitution.

Interestingly, Nb-doped samples generally exhibit increased conductivity, which is attributed to the generation of additional oxide vacancies resulting from this aliovalent substitution (Figure S4 and Table S3). This effect is more pronounced at lower temperatures and in wet atmospheres, where the ionic conductivity (oxide and proton) dominates due to the enhanced ability of the increased number of oxide vacancies to incorporate water. For example, in the case of La_2.7_Pr_2.7__Q and Pr_5.4__Q, the conductivity values in wet N_2_ at 400 °C increase from 3.0 and 15.3 mS cm^−1^, respectively, to 3.6 and 16.3 mS cm^−1^ for the corresponding Nb-doped samples under the same conditions, further demonstrating the beneficial effect of Nb-doping [24,30,43,44].

Under reducing conditions, a different behaviour was observed, with a significant drop in conductivity for the praseodymium-containing samples. This is attributed to the nearly complete reduction in Pr^4+^ to Pr^3+^, resulting in a significant decrease in the electronic conductivity. Additionally, the conductivity decreases with the increasing Pr content, with values of 5.0, 2.3, and 1.6 mS cm^−1^ for La_5.4__Q, La_2.7_Pr_2.7__Q, and Pr_5.4__Q, respectively. This decrease is primarily attributed to the shrinkage of the unit cell volume caused by the size difference between La^3+^ (1.16 Å) and Pr^3+^ (1.13 Å), which hinders the ionic conduction pathways. This volume shrinkage effect is also reflected in the slightly higher activation energies. In particular, La_5.4__Q has an activation energy of 0.70(1) eV, while La_2.7_Pr_2.7__Q and Pr_5.4__Q exhibit values of 0.79(1) and 0.76(1) eV, respectively. Furthermore, Nb-doping enhances the electrical properties under reducing conditions, particularly at high temperatures. For instance, conductivity values for Pr_5.4__Q and Pr_5.4_Nb_0.1__Q are 1.6 and 3.5 mS cm^−1^, respectively, at 700 °C (Table S3) [24,30,43,44]. It is also worth noting that a significant drop in electronic conductivity for samples with a high Pr content may be less relevant in fuel cells and electrolyzer operational conditions, where the membranes typically operate under an oxygen concentration gradient.

## 4. Conclusions

For the first time, lanthanum and praseodymium molybdates with the general formula La_5.4−x_Pr_x_Mo_1−y_Nb_y_O_12−δ_ (x = 1.35, 2.7, 4.05, 5.4; y = 0, 0.1) were successfully synthesized using the freeze-drying precursor method. The compounds were sintered at 1500 °C and subsequently cooled at different rates. The quenched samples exhibited cubic symmetry due to the inherent disorder in the crystal lattice at high temperatures. Slower cooling rates, combined with a high Pr content, resulted in cubic symmetry due to the minor distortion induced by the smaller ionic radius of Pr compared to La. In contrast, compositions with a lower Pr content stabilized the R1 rhombohedral polymorph. However, TEM studies indicate a more complex local structure depending on the Pr content, with a gradual transformation from a defect-free cubic fluorite structure at a high Pr content to one with numerous local defects and finally, a modulated arrangement or domains embedded in the cubic phase at a low Pr content.

Scanning electron microscopy (SEM) and energy-dispersive X-ray spectroscopy (EDS) analyses revealed that the synthesized samples are dense and exhibit a homogeneous cation distribution, regardless of the preparation conditions or the crystal structure adopted.

The highest conductivity values were observed in a N_2_ atmosphere, which can be attributed to the presence of mixed Pr^4+^/Pr^3+^ cations. However, the proton conductivity decreased due to an increase in the p-type electronic contribution. Furthermore, in a reducing atmosphere (5% H_2_-Ar), the conductivity of Pr-substituted samples drastically decreased due to the reduction in Pr^4+^ to Pr^3^, highlighting the importance of the material composition and operating atmospheres on the conducting properties of these materials for different electrochemical applications.

## Data Availability

The original contributions presented in this study are included in the article/Supplementary Materials. Further inquiries can be directed to the corresponding authors.

## Supplementary Materials

The following supporting information can be downloaded at https://www.mdpi.com/article/10.3390/ma18030529/s1. Figure S1: XRD patterns for La_5.4−x_Pr_x_Mo_1−y_Nb_y_O_12−δ_ samples (x = 0, 1.35, 2.7, 4.05, 5.4; y = 0, 0.1) heated at 1500 °C and cooled down by quenching. The symmetry of the samples is labelled within the figure; Figure S2: XPS spectra of (a,b) O 1*s* and (c,d) La 3*d*_5/2_ core levels for the La_5.4−x_Pr_x_Mo_1−y_Nb_y_O_12−δ_ (x = 0, 1.35, 2.7. 4.05, 5.4; y = 0, 0.1) series; Figure S3: SEM micrograph and EDS mappings for La, Pr, and Mo for the La_2.7_Pr_2.7_MoO_12−δ_Q sample; Figure S4: Arrhenius plots of La_5.4−x_Pr_x_Mo_0.9_Nb_0.1_O_12−δ_ samples (x = 0, 2.7. 5.4) sintered at 1500 °C for 1 h and cooled down by quenching under dry N_2_ and wet 5% H_2_-Ar atmospheres. Table S1: Polymorphic phases, unit cell parameters, and agreement factors for La_5.4−x_Pr_x_Mo_1−y_Nb_y_O_12−δ_, (x= 0, 1.35, 2.7, 4.05, 5.4; y = 0, 0.1) sintered at 1500 °C and cooled down at different rates (quenching, 5 and 0.5 °C min^−1^); Table S2: Pr^4+^/Pr^3+^ ratio determined by the XPS of quenched samples from the La_5.4−x_Pr_x_Mo_1−y_Nb_y_O_12−δ_ series; Table S3: Conductivity values at 700 and 400 °C for La_5.4−x_Pr_x_Mo_1−y_Nb_y_O_12−δ_, (x = 0, 1.35, 2.7, 4.05, 5.4; y = 0, 0.1) sintered at 1500 °C and cooled down by quenching, under dry and wet N_2_ and wet 5% H_2_-Ar atmospheres.
