# Supplementary material for: Structure and Mixed Proton–Electronic Conductivity in Pr and Nb-Substituted La5.4MoO12−δ Ceramics
Source: Materials (Basel). 2025 Jan 24;18(3):529. doi: 10.3390/ma18030529 (PMC11818200; doi:10.3390/ma18030529)

materials

MDPI

Supplementary information

Structure and mixed proton–electronic conductivity in Pr-substituted $La_{5.4}MoO_{12-\delta}$ ceramics

Abraham Sánchez-Caballero [1], José M. Porras-Vázquez [1*], Lucía dos Santos-Gómez [1], Javier Zamudio-García [2], Antonia Infantes-Molina [1], Jesús Canales-Vázquez [3], Enrique R. Losilla [1], David Marrero-López [4*]

[1] *Universidad de Málaga, Dpto. de Química Inorgánica, Cristalografía y Mineralogía 29071-Málaga, Spain; josema@uma.es*
[2] *Department of Energy Conversion and Storage, Technical University of Denmark, Fysikvej, 310, 2800, Kgs., Lyngby, Denmark*
[3] *Renewable Energy Research Institute, Escuela Técnica Superior de Ingenieros Industriales de Albacete, University of Castilla-La Mancha, 02071, Albacete, Spain*
[4] *Universidad de Málaga, Dpto. de Física Aplicada I, 29071-Málaga, Spain; marrero@uma.es*

Table S1. Polymorphic phases, unit cell parameters, and agreement factors for $La_{5.4-x}Pr_xMo_{1-y}Nb_yO_{12-\delta}$, (x= 0, 1.35, 2.7, 4.05, 5.4; y= 0, 0.1) sintered at 1500 ºC and cooled down at different rates (quenching, 5 and 0.5 ºC min^{-1}).

	Cooling rate (ºC min^{-1})	Symmetry	a (Å)	c (Å)	V/Z (Å ^{3})	R_{wp}(%)	R_F(%)
	Q	Cubic	5.6676(1)	-	45.51(1)	6.87	3.15
**$La_{5.4}$[21]**	50	R1	3.9914(1)	9.8715(2)	45.40(1)	10.35	-
	0.5	R2	4.0996(1)	9.5204(2)	46.19(1)	9.39	-
	Q	Cubic	5.6615(1)	-	45.36(1)	5.13	2.53
**$La_{5.4}Nb_{0.1}$[24]**	50	R1	3.9908(1)	9.8989(2)	45.51(1)	7.91	-
	0.5	R1	3.9873(1)	9.9007(1)	45.44(1)	10.43	-
	Q	Cubic	5.6312(1)	-	44.64(1)	7.75	2.20
**$La_{4.05}Pr_{1.35}$**	5	Cubic	5.6187(4)	-	44.35(1)	8.34	2.52
	0.5	R1	3.9647(2)	9.8192(6)	44.56(1)	8.92	-
	Q	Cubic	5.6239(1)	-	44.47(1)	8.04	2.98
**$La_{4.05}Pr_{1.35}Nb_{0.1}$**	5	Cubic	5.6145(1)	-	44.24(1)	8.35	2.08
	0.5	R1	3.9589(1)	9.8164(6)	44.41(1)	10.36	-
	Q	Cubic	5.5986(1)	-	43.87(1)	7.62	1.78
**$La_{2.7}Pr_{2.7}$**	5	Cubic	5.5856(1)	-	43.57(1)	7.28	1.92
	0.5	Cubic	5.5864(3)	-	43.58(1)	9.29	3.22
	Q	Cubic	5.5902(2)	-	43.67(1)	7.93	1.58
**$La_{2.7}Pr_{2.7}Nb_{0.1}$**	5	Cubic	5.5777(1)	-	43.38(1)	7.25	3.07
	0.5	Cubic	5.5770(1)	-	43.36(1)	7.04	2.52
	Q	Cubic	5.5735(1)	-	43.29(1)	12.77	3.82
**$La_{1.35}Pr_{4.05}$**	5	Cubic	5.5572(1)	-	42.90(1)	8.86	3.39
	0.5	Cubic	5.5561(1)	-	42.88(1)	9.38	2.11
	Q	Cubic	5.5638(1)	-	43.06(1)	12.86	4.98
**$La_{1.35}Pr_{4.05}Nb_{0.1}$**	5	Cubic	5.5475(1)	-	42.68(1)	8.60	2.68
	0.5	Cubic	5.5461(1)	-	42.65(1)	8.83	3.17
**$Pr_{5.4}$**	Q	Cubic	5.5402(1)	-	42.51(1)	7.04	2.52

	5	Cubic	5.5267(1)	-	42.20(1)	7.22	2.95
	0.5	Cubic	5.5266(1)	-	42.20(1)	7.61	2.91
	Q	Cubic	5.5326(1)	-	42.34(1)	9.14	1.67
**$Pr_{5.4}Nb_{0.1}$**	5	Cubic	5.5190(1)	-	42.03(1)	9.04	1.45
	0.5	Cubic	5.5174(1)	-	41.99(1)	7.22	2.48

Table S2. Pr^{4+}/Pr^{3+} ratio determined by the XPS of quenched samples from the $La_{5.4-x}Pr_xMo_{1-y}Nb_yO_{12-\delta}$ series.

Sample	Pr^{4+}/Pr^{3+} ratio
**$La_{4.05}Pr_{1.35}$**	0.74
**$La_{2.7}Pr_{2.7}$**	0.53
**$La_{1.35}Pr_{4.05}$**	0.53
**$Pr_{5.4}$**	0.48
**$La_{4.05}Pr_{1.35}Nb_{0.1}$**	0.69
**$La_{2.7}Pr_{2.7}Nb_{0.1}$**	0.53
**$La_{1.35}Pr_{4.05}Nb_{0.1}$**	0.55
**$Pr_{5.4}Nb_{0.1}$**	0.49

Table S3. Conductivity values at 700 and 400 ºC for $La_{5.4-x}Pr_xMo_{1-y}Nb_yO_{12-\delta}$, (x= 0, 1.35, 2.7, 4.05, 5.4; y= 0, 0.1) sintered at 1500 ºC and cooled down by quenching, under dry and wet N_2 and wet 5% H_2-Ar atmospheres.

Sample	σ (mS cm^{-1}) dry N_2		σ (mS cm^{-1}) wet N_2		σ (mS cm^{-1}) wet 5% H_2-Ar	
	700 ºC	400 ºC	700 ºC	400 ºC	700 ºC	400 ºC
**$La_{5.4}$_Q**	1.7 10^{-1}	2.3 10^{-3}	2.5 10^{-1}	1.1 10^{-2}	5.0	2.0·10^{-1}
**$La_{5.4}Nb_{0.1}$_Q**	4.4 10^{-1}	4.0 10^{-3}	5.1 10^{-1}	2.3 10^{-2}	5.1	2.7 10^{-1}
**$La_{4.05}Pr_{1.35}$_Q**	5.5	3.1 10^{-1}	4.7	2.5 10^{-1}	1.6	3.6 10^{-2}
**$La_{4.05}Pr_{1.35}Nb_{0.1}$_Q**	5.6	3.2 10^{-1}	4.7	2.7 10^{-1}	1.7	1.8 10^{-2}
**$La_{2.7}Pr_{2.7}$_Q**	38.5	4.1	31.7	3.0	2.3	5.4 10^{-2}
**$La_{2.7}Pr_{2.7}Nb_{0.1}$_Q**	32.1	3.5	34.5	3.6	1.5	3.1 10^{-2}
**$La_{1.35}Pr_{4.05}$_Q**	97.1	12.9	76.1	8.2	3.1	7.7 10^{-2}
**$La_{1.35}Pr_{4.05}Nb_{0.1}$_Q**	97.3	13.2	83.1	8.5	3.1	7.1 10^{-2}
**$Pr_{5.4}$_Q**	204.4	25.9	111.6	15.3	1.6	3.5 10^{-2}
**$Pr_{5.4}Nb_{0.1}$_Q**	148.2	30.2	123.1	16.3	3.5	1.1 10^{-1}

Figure S1. XRD patterns for $La_{5.4-x}Pr_xMo_{1-y}Nb_yO_{12-\delta}$ samples ($La_{5.4-x}Pr_xNb_y$) (x = 0, 1.35, 2.7. 4.05, 5.4; y = 0, 0.1) heated at 1500 °C and cooled down by quenching. The symmetry of the samples is labelled is displayed within the figure.

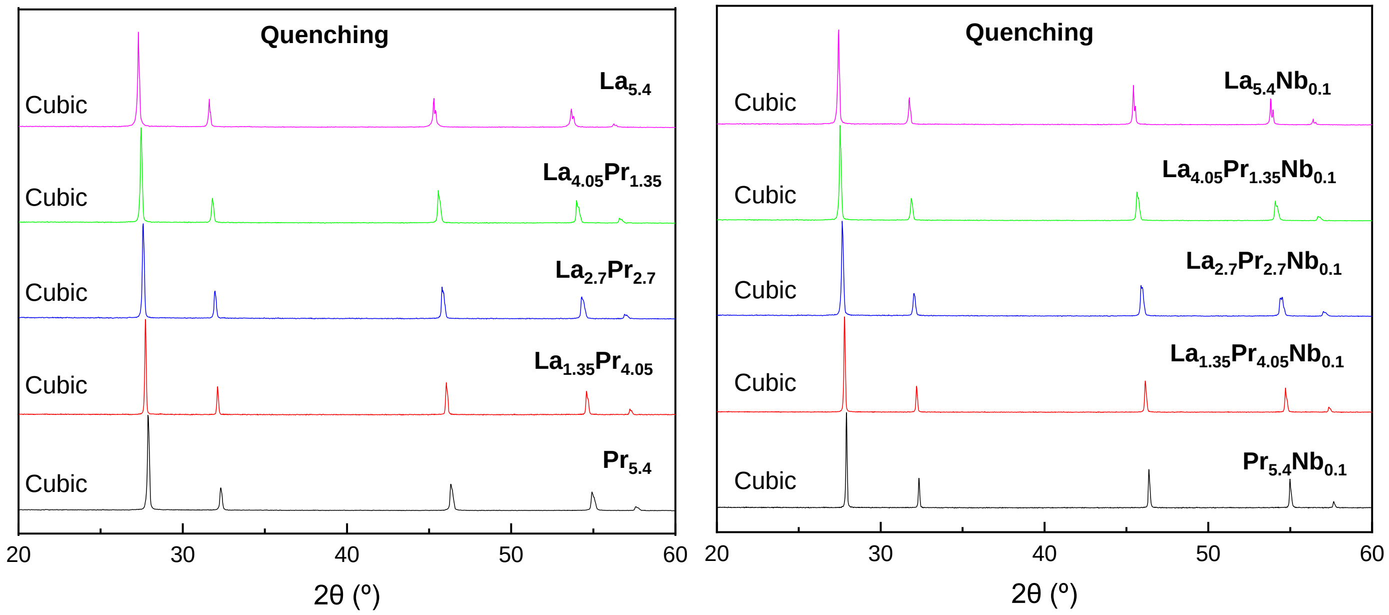
Quenching
La5.4
La4.05Pr1.35
La2.7Pr2.7
La1.35Pr4.05
Pr5.4
Cubic
20
30
40
50
60
2θ (°)
Quenching
La5.4Nb0.1
La4.05Pr1.35Nb0.1
La2.7Pr2.7Nb0.1
La1.35Pr4.05Nb0.1
Pr5.4Nb0.1
Cubic
20
30
40
50
60
2θ (°)

Figure S2. XPS spectra of (a,b) O 1*s* and (c,d) La 3*d*5/2 core levels for the $La_{5.4-x}Pr_xMo_{1-y}Nb_yO_{12-\delta}$ (x = 0, 1.35, 2.7. 4.05, 5.4; y = 0, 0.1) series.

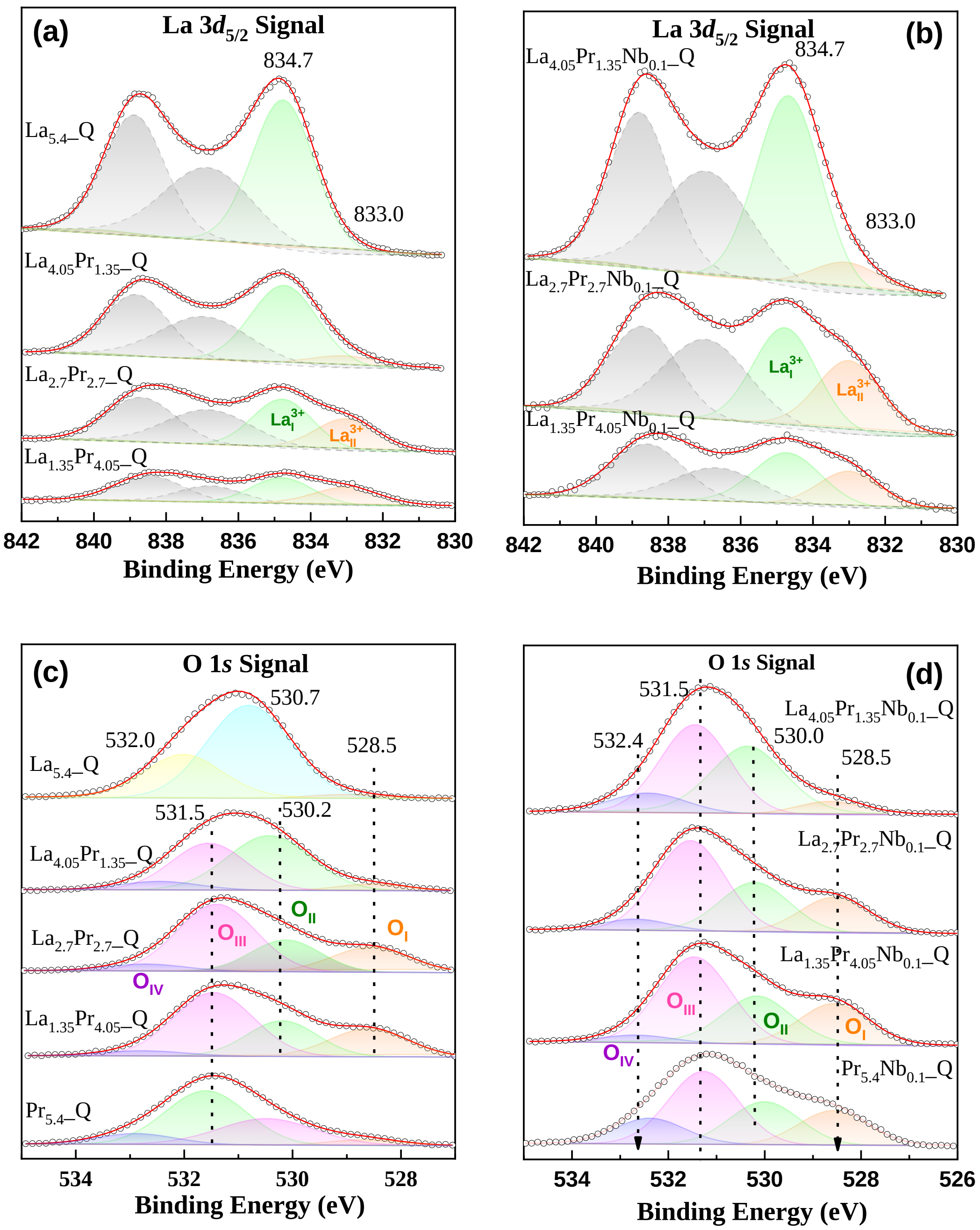

Figure S3. SEM micrograph and EDS mappings for La, Pr, and Mo for the $La_{2.7}Pr_{2.7}$**MoO**$_{12-\delta}$_Q sample.

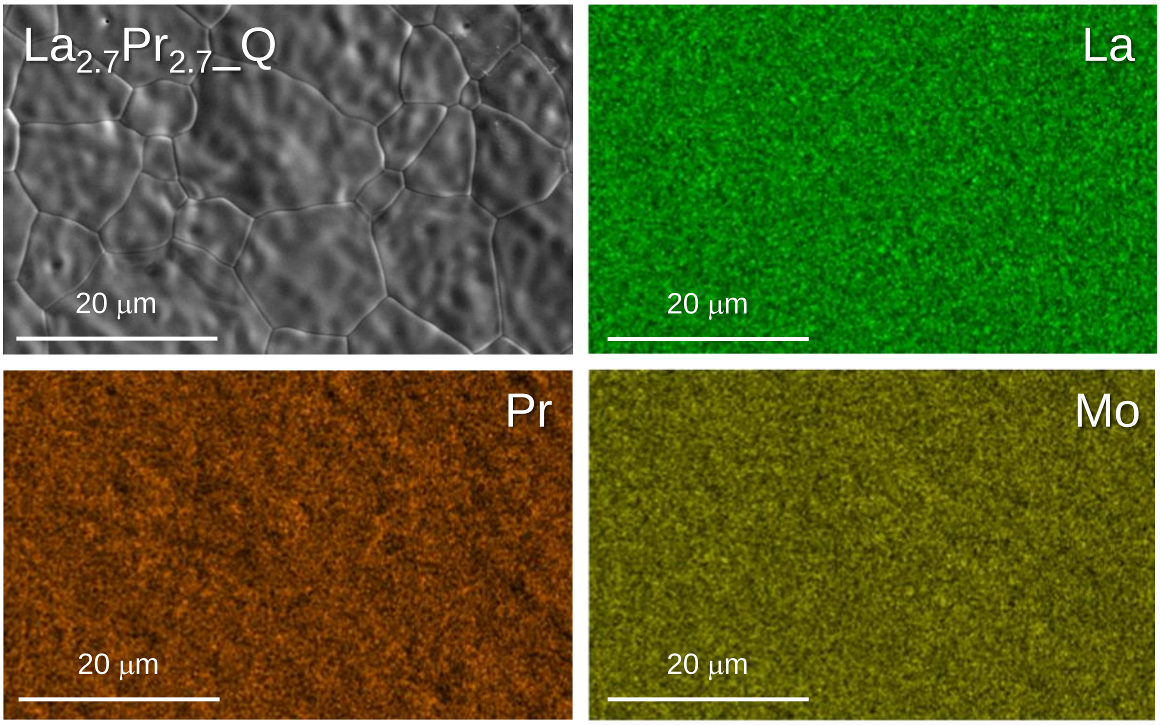

Figure S4. Arrhenius plots of $La_{5.4-x}Pr_xMo_{0.9}Nb_{0.1}O_{12-\delta}$ samples (x = 0, 2.7. 5.4) sintered at 1500 ºC for 1 h and cooled down by quenching under dry N_2 and wet 5% H_2-Ar atmospheres.

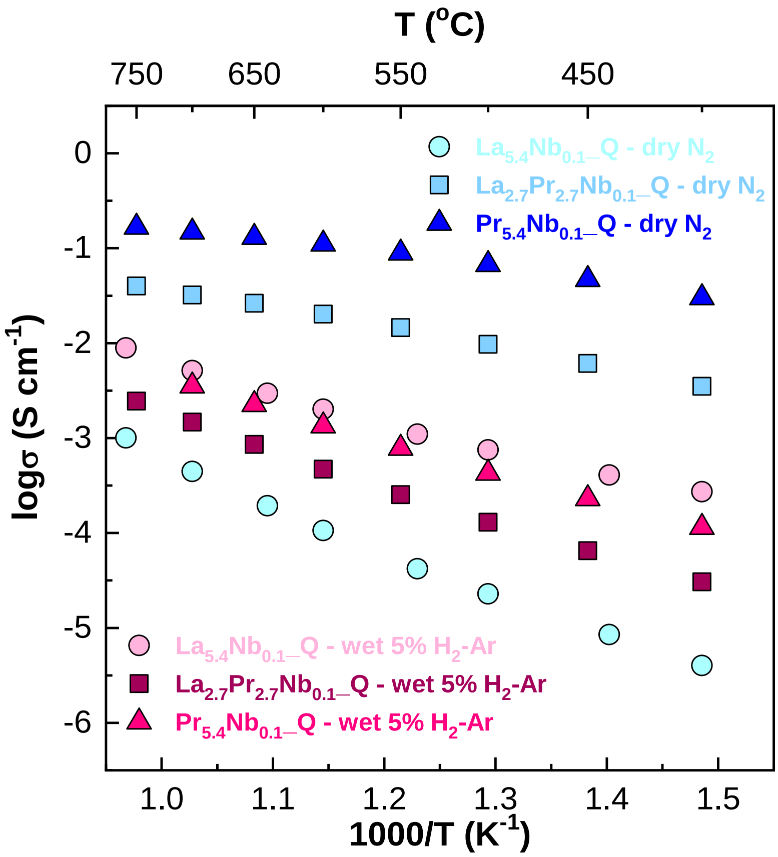

Supplement: Supplementary file 1 [file materials-18-00529-s001.zip › materials-3421885-supplementary.pdf]
